# Findings of the literature review on larviciding in elimination environments in Asia Pacific

**DOI:** 10.1186/1475-2875-11-S1-P103

**Published:** 2012-10-15

**Authors:** Maxine Whittaker, Moh Seng Chang, Goodluck Tesha

**Affiliations:** 1 Asia Pacific Malaria Elimination Network (APMEN), Queensland, Australia; 2 Australian Centre for International and Tropical Health, University of Queensland, Australia

## Background

The Vector Control Working Group of Asia Pacific Malaria Elimination Network has been working with countries to identify vector control capacities, needs and activities to assist the sharing of knowledge between partner countries.

## Materials and methods

A comprehensive literature review and A survey of malaria vector control was conducted in early 2011 amongst the then eleven APMEN countries, of these eight countries responded. The survey was conducted through a questionnaire sent via email to all APMEN Country Partner representatives at that time. The literature review addressed the questions: What vector control tools are APMEN countries are using; how are these tools selected and how are they being used in the context of malaria elimination? What else is needed to eliminate the remaining low-levels of transmission? Is there any data to indicate what methods are working or not working? What are the challenges (technical and operational) facing the use of vector control interventions for elimination available in Asia-Pacific region?

## Results

The survey provided a clear definition of the vector control tools that the APMEN countries are using. It revealed how these tools were mainly selected based on their previous experience with them in malaria control and that there is little change in the ways they are used between control and elimination, except for some targeting of the intervention sites. At low-levels of transmission many countries identified the need to consider larval control and environmental management, but seemed concerned that they have limited experience with, capacity in nor guidelines and standard operating procedures to support the use of these additional vector control methods. The countries on the whole did not report on specific information that they use to measure the effectiveness of the programmes and then the adaptation of their approaches based on this information, although many detailed some of the monitoring and evaluation tools they used. More detailed follow-up with countries will be required to get more specific information in this area.

## Conclusions

There are several challenges facing the scaling up of use of vector control in elimination settings in the Asia and Pacific region. One cluster revolves around issues of operational importance regarding a method or combination of methods. Another group of challenges are the evidence base for, and access to the evidence for use of some less commonly used vector control methods in these countries, and use in a targeted way for elimination settings. A major set of challenges relate to health system capacities - commonly mention were human resources, logistics management and health information systems (including monitoring and evaluation). Common concerns for any vector control method were also about the broader engagement of community and of other sectors in vector management - areas where the health sector and government based respondents to the survey felt less experienced and equipped.

